# Identification and Characterization of Cyclic AMP Response Element-Binding Protein H Response Element in the Human Apolipoprotein A5 Gene Promoter

**DOI:** 10.1155/2013/892491

**Published:** 2013-07-17

**Authors:** Kwang Hoon Song, Ah-Yeon Park, Ji-Eun Kim, Jin Yeul Ma

**Affiliations:** ^1^KM-Based Herbal Drug Development Group, Herbal Medicine Research Division, Korea Institute of Oriental Medicine (KIOM), 483 Expo-ro, Yuseong-gu, Daejeon 305-811, Republic of Korea; ^2^KM Health Technology Research Group, Medical Research Division, Korea Institute of Oriental Medicine (KIOM), 483 Expo-ro, Yuseong-gu, Daejeon 305-811, Republic of Korea

## Abstract

The cyclic AMP response element-binding protein H (CREBH) plays important roles in hepatic lipogenesis, fatty acid oxidation, and lipolysis under metabolic stress. Here, we report CREBH as a novel regulator of human APOA5. Knockdown of endogenous CREBH expression *via* small interfering RNA resulted in the downregulation of human APOA5 mRNA expression in human hepatoma cells, HepG2. Sequence analysis suggested that putative CREBH response element (CREBHRE) is located in the human APOA5 promoter region and is highly conserved in both human and rodent. To clarify whether the human APOA5 promoter is regulated by CREBH, we analyzed the human APOA5 promoter region using a transient transfection assay and determined that transfection of CREBH induced human APOA5 promoter activity. Moreover, it was shown that CREBH directly regulated human APOA5 gene expression by binding to a unique CREBHRE located in the proximal human APOA5 promoter region, using 5′-deletion and mutagenesis of human APOA5 promoter analysis and chromatin immunoprecipitation assay. Taken together, our results demonstrated that human APOA5 is directly regulated by CREBH *via* CREBHRE and provided a new insight into the role of this liver-specific bZIP transcription factor in lipoprotein metabolism and triglyceride homeostasis.

## 1. Introduction

Elevation of triglyceride (TG) levels, hypertriglyceridemia, has been shown to be related to increased risk of cardiovascular disease [1, 2]. Therefore, it has been attempted to identify the specific genetic determinants of plasma TG levels, and a novel member of the apolipoprotein family, apolipoprotein A5 (APOA5), was identified by the comparative sequencing of the APOA1/C3/A4 gene cluster region [3]. APOA5, which is exclusively expressed in the liver, has been shown to be important in the regulation of plasma TG levels [3, 4]. It has been reported that human APOA5 gene expression was directly upregulated by several nuclear receptors, including peroxisome proliferator-activated receptor (PPAR*α*), farnesoid X-activated receptor (FXR), retinoid acid receptor-related orphan receptor (ROR*α*), hepatocyte nuclear factor 4*α* (HNF4*α*), and thyroid receptor *β* (TR*β*) [5–8]. We also recently reported that orphan nuclear receptor Nur77 is a novel regulator of human APOA5 gene expression [9].

Cyclic AMP response element-binding protein H (CREBH) was identified as a liver-specific bZIP transcription factor [10, 11]. Recent studies have demonstrated that CREBH plays a number of important roles in the hormonal regulation of hepatic gluconeogenesis under fasting or insulin-resistant conditions, as well as in hepatic lipogenesis, fatty acid oxidation, and lipolysis under metabolic stress [12–14]. Especially, it has been reported that CREBH is required for the maintenance of normal plasma TG levels, and hypomorphic or null mutations of CREBH associated with hypertriglyceridemia in human [14]. Moreover, a recent study showed that CREBH could bind to its unique response element (CREBHRE) in the target gene promoter to modulate transcription [10, 12].

Here, we report that a CREBHRE is located in the promoter of human APOA5, which is a critical region for the transcriptional regulation of the APAO5 gene by CREBH.

## 2. Materials and Methods

### 2.1. Cell Culture

HepG2 cells were obtained from the American Type Culture Collection (Manassas, VA, USA). The cells were maintained in a 1 : 1 mixture of Dulbecco's Modified Eagle's Medium and F-12 (50 : 50; Invitrogen) supplemented with 100 U/mL penicillin G/streptomycin sulfate (Invitrogen) and 10% heat-inactivated fetal bovine serum (Gibco).

### 2.2. Luciferase Reporter Constructs and Plasmid

A series of human APOA5 promoter fragments −846, −356, and −147 were constructed in the pGL3 luciferase vector as reported previously [9]. Additional deletion constructs of human APOA5 promoter (−92 and −70) were obtained by PCR from the cloned pGL3 human APOA5 promoter (−846) as a template and were cloned in pGL3 basic luciferase vector (Clontech). The following oligonucleotide primers were used for PCR: forward primer tailed with a *Kpn I* restriction site, 5′-GGT ACC TTT TGA ACT TCC ACG TGG TAT-3′ (−92) and 5′-GGT ACC TAC TCA GAG CAA TTG GTG CCA-3′ (−70); reverse primer tailed with a *Xho I* restriction site, 5′-CTC GAG AAT GCC CTC CCT TAG GAC TGT GAC-3′. Site-directed mutagenesis of the putative human APOA5 promoter CREBH response element (CREBHRE) was performed, using the oligonucleotide 5′-GGT ACC CTT CTT TTG AAC TTC C**GG GTG GT**A TTT ACT CAG A-3′(mutated bases are indicted in bold) as a mutagenic forward primer and reverse primer, 5′-CTC GAG AAT GCC CTC CCT TAG GAC TGT GAC-3′. The expression vector pcDNA3-FLAG-CREBH-N was a kind of a gift from Dr. Hueng-Sik Choi (Chonnam National University, Gwangju, Republic of Korea) [15]. The expression vectors Nur77 and HNF4*α* were as described previously [9].

### 2.3. Transient Transfection and Luciferase Reporter Assay

For the luciferase reporter assay, HepG2 cells were plated in 24-well plates 24 h before transfection with reporter or expression plasmids using Lipofectamine 2000 reagent (Invitrogen) according to the manufacturer's instructions. The total DNA used in each transfection was adjusted by adding the appropriate amount of pcDNA3 empty vector. Luciferase activity was measured using the Dual-Glo Luciferase Assay System (Promega, Madison, WI, USA) according to the manufacturer's instructions. Assays were performed in triplicate and expressed as mean ± SD.

### 2.4. Recombinant Adenovirus, RNA Isolation, and Analysis

For endogenous knockdown of CREBH expression in HepG2 cells, we applied a recombinant adenovirus system. Adenovirus for the unspecific (Ad-USi) control and CREBH RNAi (Ad-CREBHi) were obtained from Dr. Hueng-Sik CHoi (Chonnam National University, Gwangju, Republic of Korea) [16]. Recombinant adenoviruses were amplified in HEK293A cells and were purified with Adeno-X Virus Maxi Purification kit (Clontech). Virus titer was determined by Adeno-X Rapid Titer Kit (BD Biosciences). Forty-eight hours after infection with AD-USi or Ad-CREBHi, total RNA was isolated using Tri Reagent (Sigma) according to the manufacturer's instruction. Reverse-transcription reactions were performed using the High-Capacity cDNA Reverse Transcription Kit (Applied Biosystems) following the manufacturer's instructions. The temperature conditions of the Mastercycler were 10 min at 25°C, 120 min at 37°C, 5 min at 85°C, and 4°C when on hold. About 2 *μ*g of total RNA from each sample was reverse-transcribed, and aliquots of the cDNA were subjected to semiquantitative PCR to detect CREBH, APOA5, and *β*-actin (primer sequences are available on request) and real-time quantitative PCR (Q-PCR). Q-PCR was performed to detect CREBH, APOA5, peroxisome proliferator-activated receptor gamma, coactivator 1 alpha (PGC1*α*), fatty acid synthase (FASN), and carnitine palmitoyltransferase 1a (CPT1A) mRNAs, as described previously [9].

### 2.5. Immunoblotting Analysis

 Cell lysate preparation and immunoblotting analysis were performed as described previously [17]. Anti-CREBH, anti-APOA5, and anti-*β*-actin antibodies were from Santa Cruz Biotechnology (Santa Cruz, CA, USA). The immunoblots were visualized using an ECL chemiluminescence detection system (Thermo).

### 2.6. Chromatin Immunoprecipitation Assay (ChIP)

ChIP assays were performed using a ChIP Assay Kit (Upstate Cell Signaling Solutions) according to the manufacturer's instructions with modification. HepG2 cells were cross-linked in 1% formaldehyde and were then sonicated. Cell lysate solution (5%) in ChIP dilution buffer was kept aside as “input.” Anti-CREBH (Santa Cruz Biotechnology) was added to precipitate DNA-protein complexes, and nonimmune mouse IgG (Santa Cruz Biotechnology) was used as a control. A 260-bp DNA fragment (−200 to +60) containing the CREBHRE of the human APOA5 promoter was PCR amplified for 30 cycles and was then analyzed on a 1.5% agarose gel. PCR primers for amplification were as follows: forward primer, 5′-AGC ACT TCT CTA CTG GGG CAG-3′, reverse primer, 5′-TGC CCT CCC TTA GGA CTG TGA-3′. As a negative control, PCR amplification of the human GAPDH promoter was performed using forward (5′-ATGGTTGCCACTGGGGATCT-3′) and reverse (5′-TGCCAAAGCCTAGGGGAAGA-3′) primers.

### 2.7. Statistical Analysis

All experimental data are shown as means values ± SD. Multiple groups were tested by one-way ANOVA, and Dunnett's multiple comparison test was used to determine which groups were significantly different from the control group. A *P* value < 0.05 was considered to be significant. *P < 0.05; **P < 0.001.

## 3. Results and Discussion

### 3.1. Knockdown of CREBH Decreases APOA5 Expression in HepG2 Cells

**Figure 1:**
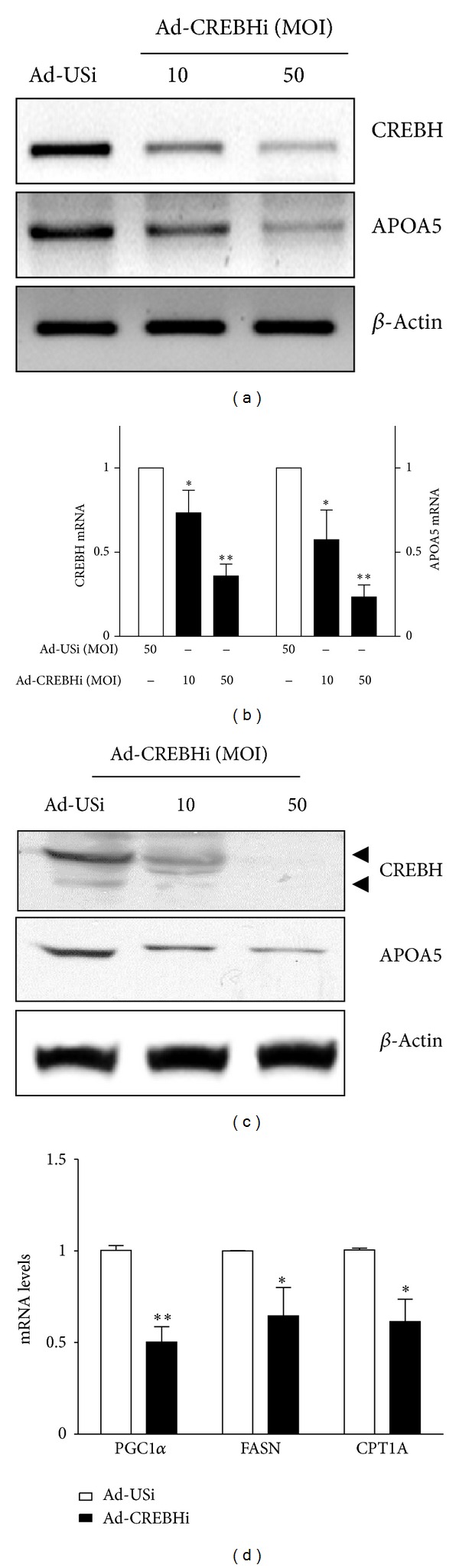
Knockdown of CREBH expression attenuates APOA5 mRNA expression in HepG2 Cells. (a) HepG2 cells were infected with adenovirus USi (Ad-USi, 50 multiplicity of infection, MOI) or CREBH shRNA (Ad-CREBHi, 10 and 50 MOI) for 48 h. Total RNA was isolated from cells for semiquantitative PCR analysis of human CREBH and APOA5 mRNA levels and were normalized to *β*-actin expression. All experiments were performed in triplicate, and data represent one of three separate experiments. (b) Total RNA was isolated for Q-PCR analysis of CREBH and APOA5 mRNA levels. Data show the relative mRNA expression of CREBH shRNA (CREBHi, 10 and 50 MOI) treated samples compared to the control USi (Ad-USi, 50 MOI) treated samples. The *(*P* < 0.05) and **(*P* < 0.01) indicate statistically significant differences between CREBHi and USi. (c) The effects of siRNAs on CREBH and APOA5 expressions were measured by western blot analysis. *β*-actin expression was used as a loading control. Arrow-heads indicate the CREBH full length (upper) and CREBH active form (lower). (d) Gene expression analysis involved in lipogenesis, triglyceride synthesis, and fatty acid oxidation. Total RNAs from HepG2 cells infected with Ad-USi (50 MOI, open bar) or Ad-CREBHi (50 MOI, filled bar) were subjected to Q-PCR analysis of gene expression. mRNA levels are shown by comparing to the HepG2 cells infected with Ad-USi. *(*P* < 0.05) and **(*P* < 0.01) indicate statistically significant difference between CREBHi and USi.

Recent studies have suggested that the hepatocyte specific transcription factor CREBH is required for the maintenance of normal plasma triglyceride [13, 14]. In addition, it has been demonstrated that APOA5 plays an important physiological role in the regulation of plasma triglyceride homeostasis [3, 18]. On the basis of those observations, we addressed the role of CREBH in APOA5 gene expression in the human hepatoma cell line, HepG2, using the adenoviral-mediated knockdown of CREBH expression. Knockdown of CREBH led to a significant reduction of APOA5 mRNA levels in HepG2 cells, demonstrating that CREBH plays an important role in the regulation of human APOA5 gene expression in human hepatoma cells (Figure 1(a)). Q-PCR and immunoblotting analysis confirmed that CREBHi infection decreased CREBH and APOA5 mRNA expressions (Figure 1(b)) and protein expression (Figure 1(c)). Consistent with the observed lower expression of APOA5 in *CrebH* null mice than in wild-type mice, our result provides further evidence that the hepatic expression of APOA5 is dependent on CREBH. To further delineate the physiological meaning of the APOA5 gene regulation by CREBH, we performed Q-PCR analysis of lipid metabolism-involved genes, using RNA isolated from CREBHi-infected HepG2 cells, which were confirmed to exhibit a reduced expression of APOA5 mRNA (Figure 1(a)–1(c)). Q-PCR analysis showed that knockdown of CREBH in HepG2 cells significantly downregulated the expression of PGC1*α*, FASN, and CPT1A mRNA, which are involved in lipogenesis and fatty acid oxidation (Figure 1(d)). These results further support the involvement of CREBH not only in APOA5 gene expression but also in maintaining hepatic lipid homeostasis.

### 3.2. Identification of CREBHRE in Human APOA5 Gene Promoter

**Figure 2:**
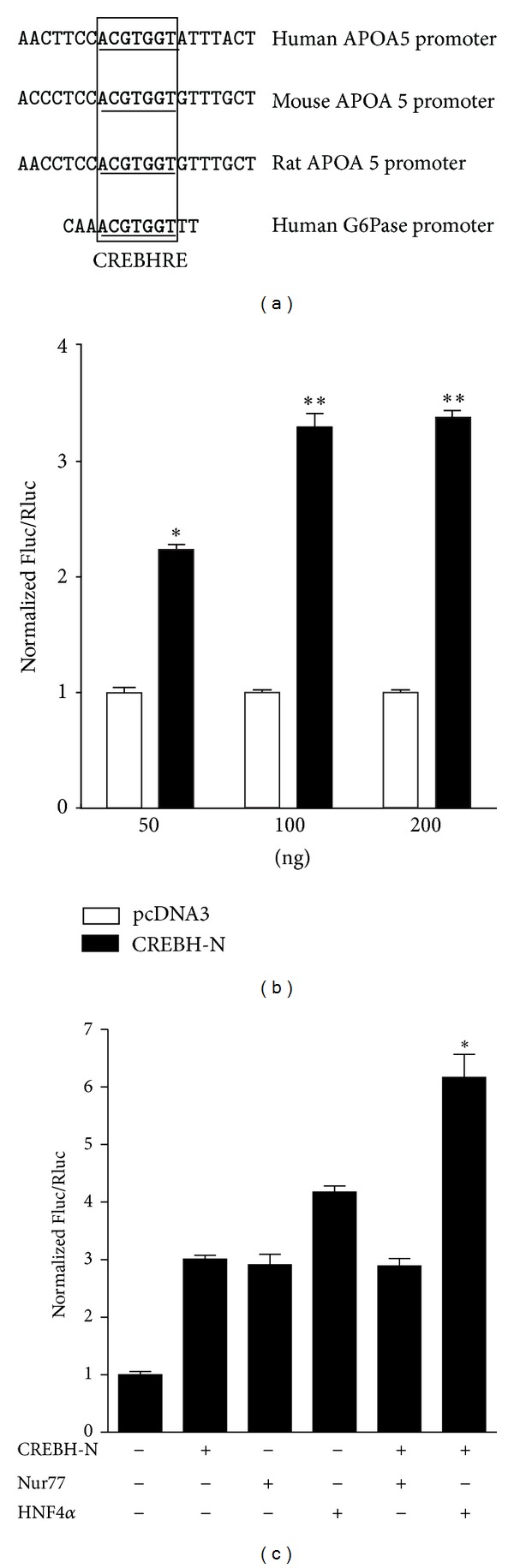
Induction of human APOA5 gene promoter activity by CREBH-N. (a) Alignment of the putative CREBHRE on APOA5 promoter in human, mouse, rat, and human G6Pase promoter. Bold and box indicate a high degree of sequence conservation within the putative CREBHRE between human and rodent. (b) HepG2 cells were cotransfected with the human APOA5 promoter (−846/+62) along with increasing amounts of CREBH-N (50, 100, and 200 ng) or empty plasmid pcDNA3 as a control. (c) HepG2 cells were cotransfected with the human APOA5 promoter (−846/+62, 200 ng) along with the CREBH-N expression plasmid (100 ng) and Nur77 or HNF4*α* expression plasmid (100 ng). The *(*P* < 0.05) indicates statistically significant differences of CREBH alone or HNF4*α* alone versus CREBH with HNF4*α*. Luciferase activity was measured using the Dual-Glo Luciferase Assay System. The data are represented as the ratio of firefly luciferase (Fluc) activity to Renilla luciferase (Rluc) activity. All experiments were performed in triplicate and data represent mean values ± SD of three individual experiments. Statistical differences from controls are indicated by asterisks (*P < 0.05 and **P < 0.01).

Data base analysis revealed the existence of the consensus CREBH response element, the ACGTGGT sequence, at the −81/−75-bp region of human APOA5 promoter. The identification of a CREBH binding site in the human APOA5 promoter led us to examine the cross-species conservation of this sequence.  Figure 2(a) shows the sequence alignment of the orthologous rat, mouse, and human APOA5 promoter sequences. The CREBHRE in the human promoter at positions −81 to −75 was highly conserved in both mouse and rat sequences. Interestingly, our mapping study showed that the putative CBREHRE sequences in the human APOA5 promoter are perfectly matched with the recently identified CREBH binding site in the human G6Pase promoter [12].

To examine whether CREBH regulates human APOA5 gene expression, transient transfection-based reporter luciferase assays were performed in HepG2. Since CREBH was shown to be activated via enhanced expression and increased proteolytic processing, to produce an active nuclear moiety which resulted in the accumulation of an active nuclear form of CREBH (CREBH-N) [12], we used CREBH-N in the transient transfection assay. As predicted, cotransfection of the active form of CREBH (CREBH-N) significantly and dose dependently activated promoter activity of human APOA5, compared with the vehicle control (Figure 2(b)). These observations are consistent with the concept that CREBH enhances human APOA5 gene expression directly, by binding CREBHRE in their promoter regulatory region, and further confirms that this induction requires the activation function of CREBH.

Considering the fact that it has been previously reported that Nur77 and HNF4*α* are involved in APOA5 gene expression [8, 9], it is also possible that CREBH functions on APOA5 gene expression, together with these transcription factors. We investigated whether CREBH physically interacts with these transcription factors via coimmunoprecipitation assay, but no significant interaction was observed between CREBH and Nur77 or HNF4*α* (data not shown). Although the fact that CREBH does not physically interact with Nur77 or HNF4*α*, the CREBHRE is close to Nur77 response element and HNF4*α* response element in the APOA5 promoter, suggesting that coactivation occurred between CREBH and Nur77 or HNF4*α*. Interestingly, the simultaneous cotransfection of CREBH and HNF4*α* caused a dramatic activation of APOA5 promoter activity, compared to that when either factor alone was cotransfected (Figure 2(c)). However, cotransfection of CREBH and Nur77 together showed no significant increase in APOA5 promoter activity. These results suggest that CREBH functions with HNF4*α* in the regulation of APOA5 promoter activity.

**Figure 3:**
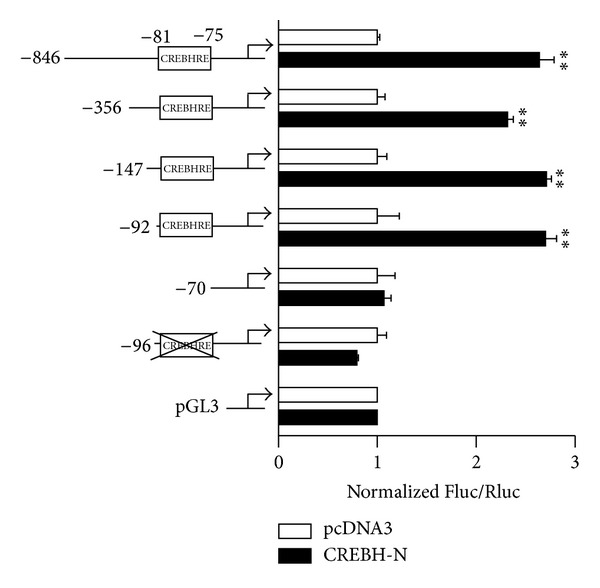
Identification of the response sequence to CREBH in the human APOA5 gene promoter. A series of deletion constructs of human APOA5 promoter luciferase reporters and a construct containing a mutation (cross) of the putative CREBH binding site (CREBHRE) were cotransfected with a plasmid expressing CREBH-N or the empty plasmid pcDNA3 as a control, into HepG2 cells. Luciferase activity was measured using the Dual-Glo Luciferase Assay System. Representative data from three independent experiments are shown. Statistical differences from controls are indicated by asterisks (*P < 0.05 and **P < 0.01).

To confirm the functionally important putative CREBHRE in the human APOA5 promoter, we generated a series of reporter constructs containing 5′ deletions and tested them using luciferase reporter assays (Figure 3). We measured the reporter activity in cells cotransfected with vectors encoding CREBH-N. Whereas the −846, −356, −147, and −92 to +62 fragments of the human APOA5 promoter were activated to similar extents by cotransfection with CREBH-N, luciferase activity of the construct containing the region from −70 to +62, which lost the CREBHRE site, was not significantly activated, even with CREBH-N (Figure 3). This result indicated that sequences between positions −92 and −70 are necessary and sufficient for CREBH-N-mediated human APOA5 promoter activity. Additional experiments with promoter fragments containing mutations in the CREBHRE position showed that this alteration had no influence on the effect of CREBH-N cotransfection on promoter reporter activity. Deletions or mutations of CREBHRE abolished the activation of the reporter by CREBH, suggesting that CREBHRE is required for the optimal activation of human APOA5 genes by CREBH. Taken together, these results indicate that the putative CREBHRE site between −81 and −75 is required for CREBH-mediated transactivation of human APOA5 promoter activity. 

Previous studies have reported that insulin-mediated repression of APOA5 gene expression is involved in the phosphorylation of upstream stimulatory factors (USFs), through the PI3K and P70 S6 kinase pathways, and resulted in the loss of their binding to the E-box element in APOA5 promoter, as well as that SREBP-1c downregulates APOA5 gene expression via E-box element, which is known as a SREBP binding site [19, 20]. Since the E-box sequence is overlapped with the CREBHRE sequence, and *CrebH* null mice showed partial repression of APOA5 mRNA, we can speculate that not only CREBH but also USF is involved in APOA5 gene regulation at the transcriptional level. Moreover, there may be some molecular link between CREBH and APOA5 gene expressions, possibly through the effects on SREBP-1c, since the role of SREBP-1c in the downregulation of APOA5 gene expression through the E-box elements located in APOA5 promoter has been demonstrated [20]. When expression of CREBH was high, such as during conditions of fasting, the transcription of APOA5 gene was synergistically activated, and SREBP-1c binding was significantly reduced, compared with where it was expressed independently. 

### 3.3. Binding of CREBH to Endogenous CREBHRE in HepG2 Cells

**Figure 4:**
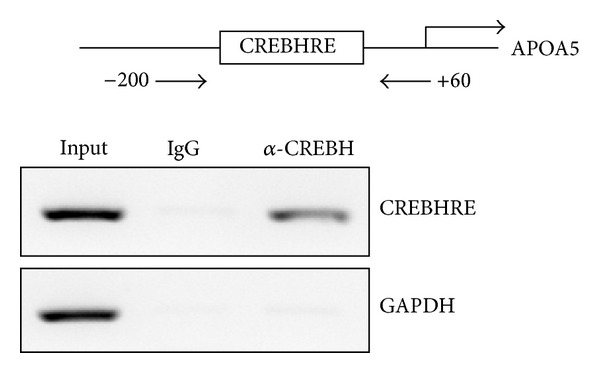
CREBH binds to APOA5 promoter. HepG2 cells were subjected to formaldehyde cross-linking, and chromatin fragments were prepared by sonication and immunoprecipitated anti-CREBH antibody. Promoter sequences containing the CREBHRE sequence were analyzed by PCR using primer sets specific to the human APOA5 promoter. As a negative control, primers were used to amplify the GAPDH promoter region which was not expected to interact with the CREBH. Cell lysate solution (5%) in ChIP dilution buffer was kept aside as “input.” Three separate experiments were carried out, and a representative example of the result is shown.

Subsequently, to confirm the binding of CREBH to the human APOA5 promoter, ChIP assays were carried out (Figure 4). PCR amplification of a region containing CREBHRE indicated the accumulation of CREBH at the promoter, whereas no significant recruitment of CREBH was observed at the GAPDH promoter, which served as a negative control. In addition, no PCR amplification was observed when immunoprecipitations assay were carried out using mouse IgG. Our ChIP assay showed a positive signal in the region from −81 to −75, indicating that CREBH protein complex is formed in the CREBHRE region *in vitro*. Together with deletion and site-directed mutagenesis, binding analysis data are in agreement with the concept that CREBH regulates human APOA5 gene expression directly, via its unique response element CREBHRE.

## 4. Conclusions

In conclusion, we provide evidence that the human APOA5 gene is a novel target of the liver-enriched endoplasmic reticulum-bound transcription factor and demonstrate that CREBH binds to the specific response element in the human APOA5 promoter and participates in the regulation of human APOA5 gene expression in human hepatoma cells.
